# Hypercalcemia Due to Progressive Disseminated Histoplasmosis

**DOI:** 10.1210/jcemcr/luae198

**Published:** 2024-10-22

**Authors:** Lakshmipriya Thandiyekkal Rajan, Naman Aggarwal, Jayakrishnan C Menon, Subhash Yadav, Rungmei S K Marak

**Affiliations:** Department of Endocrinology, Sanjay Gandhi Post Graduate Institute of Medical Sciences, Lucknow, Uttar Pradesh 226014, India; Department of Endocrinology, Sanjay Gandhi Post Graduate Institute of Medical Sciences, Lucknow, Uttar Pradesh 226014, India; Department of Endocrinology, Sanjay Gandhi Post Graduate Institute of Medical Sciences, Lucknow, Uttar Pradesh 226014, India; Department of Endocrinology, Sanjay Gandhi Post Graduate Institute of Medical Sciences, Lucknow, Uttar Pradesh 226014, India; Department of Microbiology, Sanjay Gandhi Post Graduate Institute of Medical Sciences, Lucknow, Uttar Pradesh 226014, India

**Keywords:** hypercalcemia, histoplasmosis, granulomatous disease, 1,25-dihydroxyvitamin D, PTH-independent, amphotericin B

## Abstract

Hypercalcemia is a relatively common clinical problem, and evaluation for its etiology may often prove to be challenging. However, a thorough etiological workup can guide effective therapy and can often prove to be lifesaving. We describe a 61-year-old man who presented with fever, anorexia, and weight loss for 3 months, and altered sensorium for around 1 week. His evaluation revealed severe hypercalcemia, correction of which led to improvement in his symptoms. Workup for the cause revealed that he had parathyroid hormone–independent hypercalcemia with elevated levels of 1,25-dihydroxyvitamin D, suggesting a granulomatous disease. Radiological evaluation was suggestive of a multisystem disorder with bilateral adrenal enlargement, generalized lymphadenopathy, and hepatosplenomegaly. Biopsy from the adrenal gland and bone marrow clinched the diagnosis of progressive disseminated histoplasmosis, which required treatment with liposomal amphotericin B for a total duration of 4 weeks, followed by oral itraconazole. The effective treatment was associated with normalization of serum calcium and disappearance of symptoms. Histoplasmosis represents a rare cause of hypercalcemia, with only around 22 such cases having been reported worldwide.

## Introduction

Hypercalcemia can often be a perplexing clinical problem due to the myriad ways in which it can present. The prognosis of the patient rests on making the correct etiological diagnosis and instituting appropriate treatment in a timely manner, delay of which may prove life-threatening. The etiology of hypercalcemia can be separated into 2 major categories, parathyroid hormone (PTH)-dependent and PTH-independent [1, 2]. The latter encompasses a wide variety of conditions such as various neoplasms, vitamin D toxicity, rare inherited conditions, and various granulomatous conditions like sarcoidosis and tuberculosis. In India, the most common cause of granulomatous disease–associated hypercalcemia is tuberculosis, with hypercalcemia being reported in up to 20% of cases [3]. We describe a case of disseminated histoplasmosis presenting with hypercalcemia, its treatment, and outcome.

## Case Presentation

A 61-year-old man, farmer by occupation, residing in North India, presented with history of fever, anorexia, and weight loss of around 30 kg in 3 months. Fever was high-grade (maximum recorded temperature 103 °F), intermittent, and did not remit despite multiple courses of antibiotics. Around a week prior to presentation at our center, attendants report that he became progressively drowsy and disoriented. He had been on treatment for type 2 diabetes mellitus for the past 12 years (on dapagliflozin 5 mg/day for 6 months prior to presentation) and systemic hypertension for the past 2 months (amlodipine 2.5 mg/day). Both conditions were well controlled. Glycated hemoglobin level was 6.7% on admission. Blood pressure in the hospital was persistently normal off antihypertensives, and they were subsequently discontinued. There was no history of nephrolithiasis, fractures, tuberculosis, or any other significant illness in the past. At presentation, he was drowsy and disoriented to time and place, but not to person. He was febrile, and appeared pale and dehydrated. He had mild hepatosplenomegaly.

## Diagnostic Assessment

**Figure 1.**
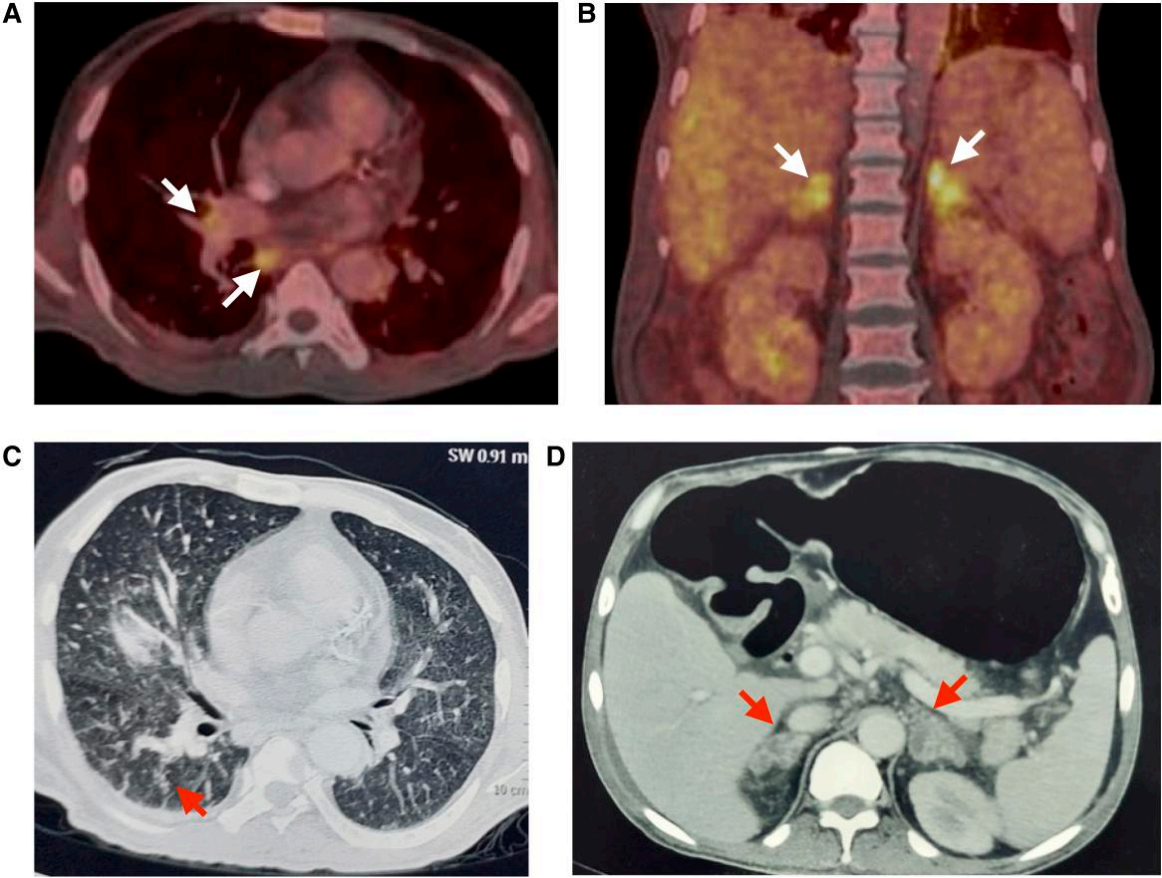
Radiological evaluation of the patient. A, Axial section of ^18^F-FDG/PET-CT scan showing FDG-avid hilar and subcarinal lymph nodes (arrows). B, Coronal section of ^18^F-FDG/PET-CT scan showing diffuse symmetric FDG avidity in bilateral adrenal glands which are enlarged (arrows) and hepatosplenomegaly. C, Axial section of CECT scan showing centrilobular nodules in right lung lower lobe (arrow). D, Axial section of CECT scan showing both adrenal glands that are enlarged with internal areas of necrosis (arrows). Abbreviations: ^18^F-FDG/PET-CT, ^18^F-fluorodeoxyglucose positron emission tomography-computed tomography; CECT, contrast-enhanced computed tomography.

**Figure 2.**
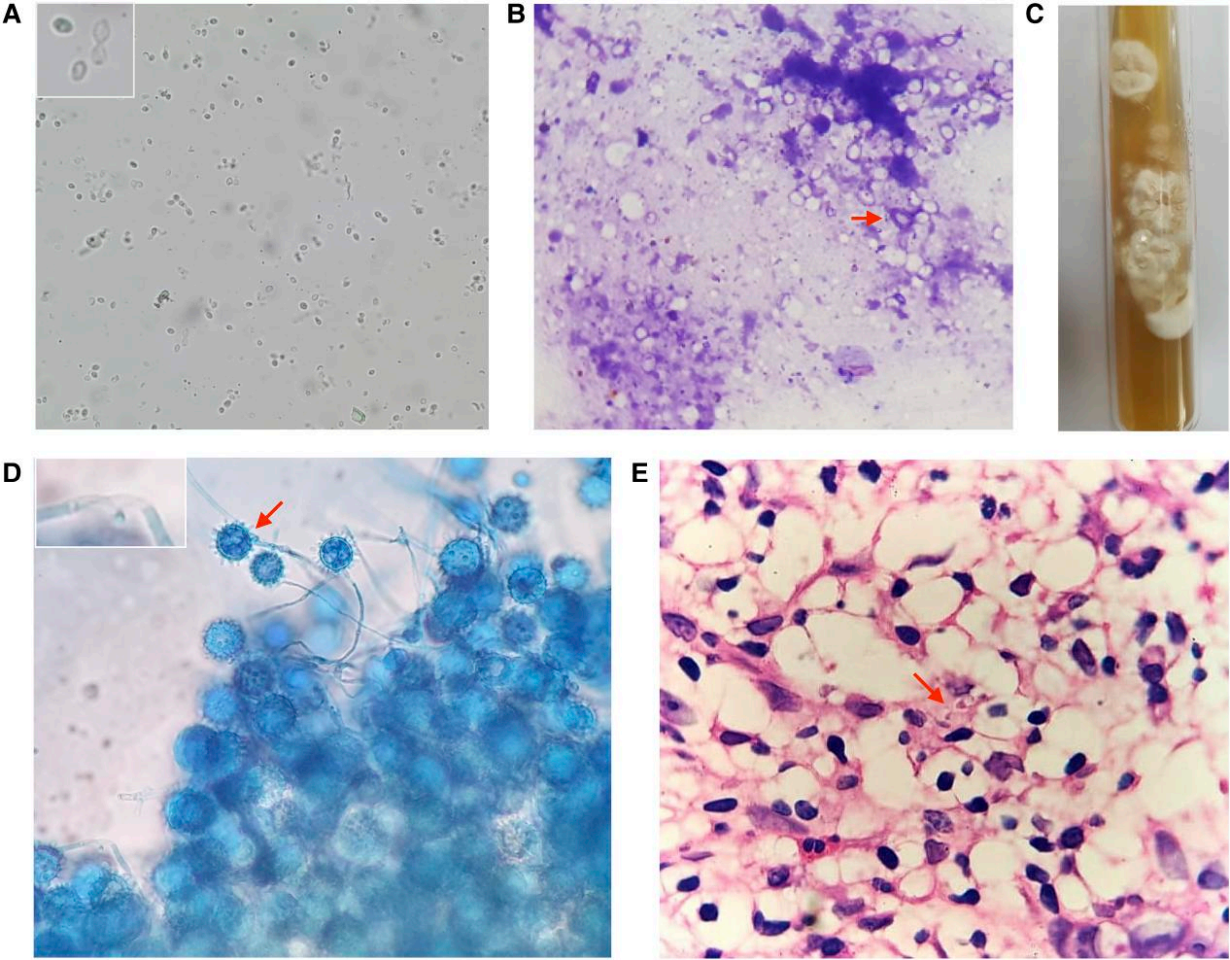
Microbiological evaluation of the patient. A, 10% KOH mount of the crushed adrenal biopsy tissue showing plenty of small, oval budding yeast cells (2-4 μm in diameter), at 40× magnification (further magnified in inset). B, Giemsa stain of the crushed biopsy tissue shows plenty of small budding yeasts where the fungal cell wall is stained blue (arrow), at 100× magnification. C, Culture on Sabouraud dextrose agar at 25°C shows growth of white mycelial colonies after 2 weeks of incubation. D, Lactophenol cotton blue stain of culture displaying plenty of tuberculate macroconidia 8 to 4 μm (arrow) and a few microconidia 2 to 4 μm in diameter (magnified in inset), at 40× magnification. E, Hematoxylin and eosin stain of the bone marrow biopsy displaying oval yeast forms with an eccentrically placed nucleus (arrow), at 100× magnification. Abbreviations: KOH, potassium hydroxide.

Biochemical parameters of the patient are provided in Table 1. He had severe hypercalcemia (serum calcium 16.9 mg/dL [4.2 mmol/L]; corrected calcium 17.9 mg/dL [4.5 mmol/L] [normal reference range, 8.5-10.8 mg/dL; 2.1-2.7 mmol/L]). Serum PTH level was 15.9 pg/mL (1.7 pmol/L) (normal reference range,15-65 pg/mL; 1.6-6.9 pmol/L), indicating PTH-independent hypercalcemia. Serum 25-hydroxyvitamin D (25OHD) level done by electrochemiluminescence immunoassay (Cobas e601, Roche Diagnostics GmbH) was 74.6 ng/mL (185 nmol/L) (normal reference range, 20-100 ng/mL; 50-250 nmol/L). Serum 1,25-dihydroxyvitamin D (1,25(OH)_2_D) by chemiluminescence immunoassay (Liaison XL, Diasorin Inc) was elevated (132.0 pg/mL [314.4 pmol/L] [normal reference range, 19.2-79.6 pg/mL; 45.6-189.6 pmol/L]). In view of raised 1,25(OH)_2_D, he was suspected to have a granulomatous disease leading to hypercalcemia. Serum angiotensin-converting enzyme level was 87 U/L (normal range, 12-68 U/L). Chest x-ray revealed bilateral consolidation. ^18^F-fluorodeoxyglucose (^18^F-FDG) positron emission tomography/computed tomography (PET-CT) showed metabolically active cervical, mediastinal, and retroperitoneal lymph nodes and bulky adrenal glands (Fig. 1). Contrast-enhanced CT of the abdomen and chest revealed bilateral adrenal gland enlargement, hepatosplenomegaly, patchy consolidation in bilateral lower lung fields, tree-in-bud pattern in the left upper lobe, and retroperitoneal and mediastinal lymph nodes with central necrosis (see Fig. 1). Adrenal insufficiency was ruled out with 8 Am serum cortisol of 12.6 μg/dL (349 nmol/L) (normal range, 6.0-18.4 μg/dL; 166-507 nmol/L), plasma adrenocorticotropin 50.8 pg/mL (11.2 pmol/L) (normal range, 7.3-63 pg/mL, 1.6-13.9 pmol/L), and plasma renin activity of less than 0.2 ng/mL/hour (normal range, 0.3-1.9 ng/mL/hour). A CT-guided adrenal biopsy was performed, and 10% potassium hydroxide (KOH) wet mount showed plenty of small budding yeasts and Giemsa stain showed yeasts with halo suggestive of histoplasma (Fig. 2). The lactophenol cotton blue staining of culture revealed plenty of tuberculate macroconidia (see Fig. 2) and was identified as *Histoplasma capsulatum*. A Cartridge Based Nucleic Acid Amplification Test (GeneXpert) performed to rule out tuberculosis was negative. Bone marrow biopsy revealed cellular marrow with trilineage hematopoiesis with granulomatous inflammation and a few capsulated organisms suggestive of histoplasmosis (see Fig. 2).

**Table 1.** Key biochemical parameters at presentation and during follow-up of the patient

Biochemical parameters	At admission	7 d after admission	At discharge	3 mo after discharge	Reference range
Calcium	**16.9 mg/dL** **(4.2 mmol/L)**	8.0 mg/dL (2.0 mmol/L)	8.1 mg/dL (2.0 mmol/L)	9.5 mg/dL (2.4 mmol/L)	8.5-10.8 mg/dL (2.1-2.7 mmol/L)
Corrected calcium	**17.9 mg/dL** **(4.5 mmol/L)**	9.5 mg/dL (2.4 mmol/L)	9.1 mg/dL (2.3 mmol/L)	9.5 mg/dL (2.4 mmol/L)	8.5-10.8 mg/dL (2.1-2.7 mmol/L)
Creatinine	0.8 mg/dL (68.9 μmol/L)	0.7 mg/dL (61.9 μmol/L)	0.7 mg/dL (61.9 μmol/L)	0.8 mg/dL (70.7 μmol/L)	0.5-1.2 mg/dL (44.2-106.1 μmol/L)
Phosphorus	**1.8 mg/dL** **(0.6 mmol/L)**	**1.9 mg/dL** **(0.6 mmol/L)**	**5.7 mg/dL** **(1.8 mmol/L)**	3.3 mg/dL (1.1 mmol/L)	2.5-4.5 mg/dL (0.8-1.5 mmol/L)
Alkaline phosphatase	**351 IU/L**	**519 IU/L**	**577 IU/L**	136 IU/L	<150 IU/L
Albumin	**2.8 g/dL** **(28.0 g/dL)**	**2.1 g/dL** **(21.0 g/dL)**	**2.7 g/dL** **(27.0 g/dL)**	4.2 g/dL (42.0 g/dL)	3.5-5.5 g/dL (35.0-55.0 g/dL)
Cortisol	12.6 μg/dL (349.2 nmol/L)	ND	25.1 μg/dL (691.6 nmol/L)	12.6 μg/dL (348.2 nmol/L)	6.0-18.4 μg/dL (166.0-507.0 nmol/L)
Parathyroid hormone	15.9 pg/mL (1.7 pmol/L)	ND	ND	ND	15.0-65.0 pg/mL (1.6-6.9 pmol/L)
25-Hydroxyvitamin D	74.6 ng/mL (185 nmol/L)	ND	ND	ND	20.0-100.0 ng/mL (50-250 nmol/L)
1,25-Dihydroxyvitamin D	**132.0 pg/mL** **(314.4 pmol/L)**	ND	ND	ND	19.2-79.6 pg/mL (45.6-189.6 pmol/L)

Abnormal values are shown in bold font. Values in parenthesis are International System of Units (SI).

Abbreviation: ND, no data.

## Treatment

Hypercalcemia was initially treated with vigorous intravenous (IV) hydration with normal saline and subcutaneous calcitonin 4 units per kilogram body weight (bw) every 12 hours. This led to improvement in sensorium. IV zoledronic acid 4 mg was given after initial investigations to keep hypercalcemia in check. His serum calcium levels normalized in 7 days. His blood glucose levels were controlled using a basal bolus insulin regimen.

After the diagnosis of progressive disseminated histoplasmosis (PDH) was made, the patient was started on IV liposomal amphotericin B at 5 mg per kg bw. His fever responded and amphotericin B was continued for 14 days. After stopping amphotericin B, he was switched to oral itraconazole 200 mg thrice daily for 3 days followed by twice daily. After 48 hours, he had recurrence of fever. Initially a catheter-related bloodstream infection was suspected and he was restarted on IV antibiotics meropenem and teicoplanin to cover for methicillin-resistant *Staphylococcus aureus* and gram-negative pathogens. He continued to have fever spikes though his blood and urine cultures were sterile. A transthoracic echocardiography was performed with a suspicion of infective endocarditis but it was negative. Serum procalcitonin was 0.23 μg/L (>0.25 μg/L indicates presence of bacterial infection). Hence, IV antifungal therapy was restarted and continued for another 14 days. This was associated with remission of fever within 48 hours, and it did not recur with subsequent stoppage. He was then transitioned to oral itraconazole 400 mg/day and discharged.

## Outcome and Follow-up

The patient was seen 1 month after discharge, and by then he had gained 14 kg of weight. He had regained his appetite and was afebrile. His biochemical parameters on follow-up are detailed in Table 1. Therapeutic drug monitoring was performed using ultra high-performance liquid chromatography, and after 1 month of oral itraconazole his serum itraconazole levels were less than the target range (0.85 μg/mL) (target range, 2-4 μg/mL). Hence the dose was increased to 200 mg thrice a day. He was last followed up 3 months after discharge from the hospital, and he continued to improve with no electrolyte imbalance, and without worsening of clinical parameters.

## Discussion

Hypercalcemia is a frequently encountered form of electrolyte imbalance, and its detection mandates workup for the underlying cause. It is commonly caused by primary hyperparathyroidism and malignancy. Rarely it can be due to elevation in 1,25(OH)_2_D, as seen in patients with granulomatous diseases, with the most frequent causes being sarcoidosis and tuberculosis, with as many as 20% of patients with tuberculosis reported to have hypercalcemia in India [3-5]. The enzyme 1α-hydroxylase in the macrophages, which mediates conversion of 25OHD to 1,25(OH)_2_D, is not under negative feedback regulation, which allows the hypercalcemia to progress unchecked [6]. Elevated or inappropriately normal 1,25(OH)_2_D in the presence of hypercalcemia is suggestive of this condition. Since ^18^F-FDG accumulates in granulomatous tissue, combined imaging with ^18^F-FDG PET-CT has proven to be a useful tool in the diagnosis and follow-up of such disorders, as seen in our case [7, 8]. Though it does not help to differentiate between etiologies like sarcoidosis, tuberculosis, or invasive fungal infections, it does help in the earlier detection of these conditions compared to planar imaging [7, 9]. The underlying cause is best determined by sampling the organ involved, if feasible.

Histoplasmosis is an infrequent etiology of PTH-independent hypercalcemia. Only about 22 cases have been reported in the literature so far [10-17]. It is usually associated with underlying immunosuppression such as with organ transplantation, HIV infection, diabetes mellitus, immunodeficiency disorders, and use of immunosuppressive drugs [18, 19]. Of the reported cases of hypercalcemia in the setting of histoplasmosis, 19 patients had disseminated infection, while 3 had pulmonary histoplasmosis. Eighteen patients survived while 4 succumbed to the illness. Histoplasmosis is endemic in the Gangetic delta, probably because soil conditions are conducive for fungal growth [20].

Conventionally it is recommended to treat PDH with IV liposomal amphotericin B for 1 to 2 weeks followed by oral itraconazole for 1 year [21]. A recent review by Galgiani and Kauffman [22] suggests that, in PDH, IV liposomal amphotericin B should be continued until clinical improvement occurs. This is exemplified in our case where IV liposomal amphotericin B had to be continued for 4 weeks due to persistence of fever, after ruling out other reasons for its occurrence. Serum itraconazole levels should be tested 2 weeks after initiation of therapy using high-performance liquid chromatography to ensure adequacy of absorption and to avoid toxicity. Maintaining levels between 2 and 4 μg per mL [22] is preferred.

The limitations in this case were that the levels of 1,25(OH)_2_D were not estimated by liquid chromatography–mass spectrometry (gold standard), as it was unavailable at our center. Also, 1α-hydroxylase expression was not studied in the biopsied tissue. Despite these limitations, the case illustrates the importance of considering histoplasmosis in the differential diagnosis for 1,25(OH)_2_D-mediated hypercalcemia, especially in endemic areas. It also illustrates the various diagnostic and therapeutic challenges encountered in the management of this rare combination.

## Learning Points

- Histoplasmosis is a rare cause of hypercalcemia due to granulomatous disease, with only around 22 cases reported in the literature to date.
- Hypercalcemia remits with appropriate antifungal therapy. Rapid diagnosis and appropriate management can be lifesaving.
- ^18^F-FDG PET/CT is invaluable in localizing sites of granulomatous inflammation and guiding tissue sampling.
- The duration of IV antifungal therapy should be individualized based on clinical response.
- It is necessary to confirm that the levels of itraconazole are in the therapeutic range after initiation of therapy.

## Data Availability

Data sharing is not applicable to this article as no data sets were generated or analyzed during this study.

## Abbreviations

^18^F-FDG/PET-CT: ^18^F-fluorodeoxyglucose positron emission tomography-computed tomography
1,25(OH)_2_D: 1,25-dihydroxyvitamin D
25OHD: 25-hydroxyvitamin D
CECT: contrast-enhanced computed tomography
IV: intravenous
KOH: potassium hydroxide
PDH: progressive disseminated histoplasmosis
PTH: parathyroid hormone

## References

[1] Renaghan AD, Rosner MH. Hypercalcemia: etiology and management. Nephrol Dial Transplant. 2018;33(4):549‐551.

[2] Ratcliffe WA, Hutchesson AC, Bundred NJ, Ratcliffe JG. Role of assays for parathyroid-hormone-related protein in investigation of hypercalcaemia. Lancet. 1992;339(8786):164‐167. 1346019 10.1016/0140-6736(92)90220-w

[3] John SM, Sagar S, Aparna JK, Joy S, Mishra AK. Risk factors for hypercalcemia in patients with tuberculosis. Int J Mycobacteriol. 2020;9(1):7‐11. 32474481 10.4103/ijmy.ijmy_211_19

[4] Donovan PJ, Sundac L, Pretorius CJ, d'Emden MC, McLeod DS. Calcitriol-mediated hypercalcemia: causes and course in 101 patients. J Clin Endocrinol Metab. 2013;98(10):4023‐4029. 23979953 10.1210/jc.2013-2016

[5] Sharma OP . Hypercalcemia in granulomatous disorders: a clinical review. Curr Opin Pulm Med. 2000;6(5):442‐447. 10958237 10.1097/00063198-200009000-00010

[6] Motlaghzadeh Y, Bilezikian JP, Sellmeyer DE. Rare causes of hypercalcemia: 2021 update. J Clin Endocrinol Metab. 2021;106(11):3113‐3128. 34240162 10.1210/clinem/dgab504

[7] Sharma P, Mukherjee A, Karunanithi S, Bal C, Kumar R. Potential role of 18F-FDG PET/CT in patients with fungal infections. AJR Am J Roentgenol. 2014;203(1):180‐189. 24951213 10.2214/AJR.13.11712

[8] Hot A, Maunoury C, Poiree S, et al Diagnostic contribution of positron emission tomography with [18F]fluorodeoxyglucose for invasive fungal infections. Clin Microbiol Infect. 2011;17(3):409‐417. 20636432 10.1111/j.1469-0691.2010.03301.x

[9] Prabhakar HB, Rabinowitz CB, Gibbons FK, O'Donnell WJ, Shepard JA, Aquino SL. Imaging features of sarcoidosis on MDCT, FDG PET, and PET/CT. AJR Am J Roentgenol. 2008;190(Suppl 3):S1‐S6. 18287458 10.2214/AJR.07.7001

[10] Alkhathlan M, Li Y, Siddiqui A, Goud S. SAT244 hypercalcemia induced by inconspicuous histoplasmosis infection. J Endocr Soc. 2023;7(Suppl 1):bvad114.540.

[11] Spiwak E, Goswami S, Lay SE, Nailescu C. Case report: histoplasmosis presenting as asymptomatic hypercalcemia detected on routine laboratory testing in a pediatric kidney transplant recipient. Front Pediatr. 2023;10:1058832. 36741088 10.3389/fped.2022.1058832 PMC9895116

[12] Gani I, Barrett A, Mulloy L, Kapoor R. Disseminated histoplasmosis presenting as weight loss and hypercalcemia in a renal transplant patient with prior history of subtotal parathyroidectomy. Am J Med Sci. 2021;361(3):383‐387. 33729918 10.1016/j.amjms.2020.09.009

[13] Rodriguez JA, Ivancic S, Eckardt PA, Lemos-Ramirez JC, Niu J. A case of pulmonary histoplasmosis presenting with hypercalcemia and altered mental status in a patient following allogeneic hematopoietic stem cell transplantation. Am J Case Rep. 2020;21:e919724. 31955178 10.12659/AJCR.919724 PMC6993276

[14] Gurram PR, Castillo NE, Esquer Garrigos Z, Vijayvargiya P, Abu Saleh OM. A dimorphic diagnosis of a pleomorphic disease: an unusual cause of hypercalcemia. Am J Med. 2020;133(11):e659‐e662. 32320694 10.1016/j.amjmed.2020.03.035

[15] Anjum A, Tumyan G. Abstract #1003234: an case of severe hypercalcemia from progressive disseminated histoplasmosis in an immunocompetent adult. Endocr Pract. 2021;27(6):S96.

[16] Agrawal S, Goyal A, Agarwal S, Khadgawat R. Hypercalcaemia, adrenal insufficiency and bilateral adrenal histoplasmosis in a middle-aged man: a diagnostic dilemma. BMJ Case Rep. 2019;12(8):e231142. 10.1136/bcr-2019-231142 PMC6720788 31466957

[17] Giordani MC, Villamil Cortez SK, Diehl M, et al Hypercalcemia as an early finding of opportunistic fungal pneumonia in renal transplantation: a case series report. Transplant Proc. 2020;52(4):1178‐1182. 32340747 10.1016/j.transproceed.2020.03.031

[18] Barros N, Wheat JL, Hage C. Pulmonary histoplasmosis: a clinical update. J Fungi (Basel). 2023;9(2):236. 36836350 10.3390/jof9020236 PMC9964986

[19] Subramanian S, Abraham OC, Rupali P, Zachariah A, Mathews MS, Mathai D. Disseminated histoplasmosis. J Assoc Physicians India. 2005;53:185‐189. 15926599

[20] De D, Nath UK. Disseminated histoplasmosis in immunocompetent individuals—not a so rare entity, in India. Mediterr J Hematol Infect Dis. 2015;7(1):e2015028. 25960856 10.4084/MJHID.2015.028 PMC4418405

[21] Wheat LJ, Freifeld AG, Kleiman MB, et al Clinical practice guidelines for the management of patients with histoplasmosis: 2007 update by the Infectious Diseases Society of America. Clin Infect Dis. 2007;45(7):807‐825. 17806045 10.1086/521259

[22] Galgiani JN, Kauffman CA. Coccidioidomycosis and histoplasmosis in immunocompetent persons. N Engl J Med. 2024;390(6):536‐547. 38324487 10.1056/NEJMra2306821

